# Comparison of the Efficacy of Topical Triamcinolone in Orabase and Curcumin in Orabase in Oral Graft-Versus-Host Disease

**Published:** 2017-11

**Authors:** Arash Mansourian, Babak Bahar, Mahdieh Sadat Moosavi, Massoud Amanlou, Shahabodin Babaeifard

**Affiliations:** 1 Associate Professor, Dental Research Center, Dentistry Research Institute, Tehran University of Medical Sciences, Tehran, Iran; Department of Oral Medicine, School of Dentistry, Tehran University of Medical Sciences, Tehran, Iran; 2 Assistant Professor, Hematology and Oncology and Stem Cell Transplantation Research Center, Tehran University of Medical Sciences, Tehran, Iran; 3 Assistant Professor, Laser Research Center of Dentistry, Dentistry Research Institute, Tehran University of Medical Sciences, Tehran, Iran; Department of Oral Medicine, School of Dentistry, Tehran University of Medical Sciences, Tehran, Iran; 4 Professor, Drug Design and Development Research Center, Department of Medicinal Chemistry, School of Pharmacy, Tehran University of Medical Sciences, Tehran, Iran; 5 Postgraduate Student, Department of Oral Medicine, School of Dentistry, Tehran University of Medical Sciences, Tehran, Iran

**Keywords:** Graft Versus Host Disease, Curcumin, Triamcinolone, Orabase

## Abstract

**Objectives:**

Graft-versus-host disease (GVHD) is among the most frequent complications of allogeneic hematopoietic stem cell transplantation (HSCT). GVHD has several clinical manifestations in the oral cavity, including painful desquamative erythema, ulcerative mucosal lesions, and lichenoid lesions. The patients presenting with oral GVHD complain of oral sensitivity, pain, dysgeusia, and xerostomia. The treatment of oral GVHD includes a proper systemic therapy combined with a good oral hygiene and the use of local and topical steroids. Corticosteroids and immunosuppressants are used for the treatment of chronic oral GVHD; however, they are associated with different complications. Evidence shows that curcumin has anti-inflammatory and antioxidative properties. The treatment of lichen planus and oral mucositis with curcumin has been successful. This study aimed to compare the efficacy of topical curcumin in Orabase and triamcinolone in Orabase in the patients affected by oral GVHD.

**Materials and Methods:**

Twenty-six patients presenting with oral GVHD were randomly divided into two groups of 13 using block randomization. The control group used triamcinolone in Orabase, and the case group received curcumin in Orabase.

**Results:**

The two groups were not significantly different in terms of the alleviated severity of the lesions at the end of the treatment (P=0.052). The comparison of the pain score via the visual analog scale (VAS) at the onset of the treatment and at days 14 and 28 (completion of the treatment) showed no significant difference between the two groups (P>0.05).

**Conclusions:**

Curcumin has comparable efficacy to that of triamcinolone and may be prescribed for the patients presenting with oral GVHD.

## INTRODUCTION

Bone marrow transplantation (BMT) is performed for the treatment of several hematologic conditions and malignancies [1, 2]. BMT is performed for the patients who cannot be treated with conventional treatment modalities [1]. Despite the optimal efficacy of BMT for the treatment of some cancers, its complications and side effects are still a matter of debate [3]. Graft-versus-host disease (GVHD) is among the most common complications of BMT, which can be fatal and has a prevalence rate of 20 to 50% [4]. GVHD is divided into two groups of acute and chronic. Acute GVHD develops three months after the transplantation and mainly involves three organs, namely the skin, gastrointestinal tract, and liver. A maculopapular rash may be seen on the palms of the hands and soles of the feet, which is itchy and/or painful [5]. Acute GVHD may affect any part of the gastrointestinal tract and has symptoms such as nausea, vomiting, stomach cramps, and diarrhea. Cholestatic jaundice is common in hepatic GVHD, but liver failure is uncommon [5]. The chronic form of GVHD may occur anytime during the first year following the transplantation. In general, the chronic GVHD symptoms do not manifest earlier than 100 days after the transplantation. In the chronic form of GVHD, the oral cavity is among the most commonly involved areas [6].

The overall prevalence rate of GVHD in the patients surviving more than six months after human leukocyte antigen (HLA)-identical BMT is about 30 to 50% [6]. According to the severity of organ involvement, two forms of chronic GVHD may be clinically observed:

1. Limited chronic GVHD: local involvement of the skin or mild liver function impairment.
2. Extensive chronic GVHD: disseminated or local involvement of the skin along with a more severe liver impairment, or involvement of the salivary glands or other organs [7].

In the past years, the incidence of acute GVHD has remained constant, while the incidence of chronic GVHD appears to be increasing [8]. Oral mucosal involvement is seen in over 80% of the patients presenting with GVHD [9]. The oral symptoms of acute GVHD include painful desquamative erythema and ulcerative mucosal lesions, while the oral symptoms of chronic GVHD may include lichenoid, erythematous, and ulcerative lesions. The patients affected by chronic GVHD also suffer from symptoms such as oral sensitivity, pain, dysgeusia, and xerostomia [4, 10]. This condition can bear some similarity with autoimmune conditions, such as lupus erythematosus, lichen planus, and Sjogren’s syndrome [11].

In 2006, the National Institute of Health (NIH) published a guideline that included parameters for scoring the severity of chronic GVHD (mild, moderate, severe) and the response to treatment in the affected oral areas [12]. Due to the nature of this condition, its treatment includes several therapeutic measures to maximize the therapeutic efficiency. The treatment modalities for oral GVHD include a proper systemic treatment in combination with a good oral hygiene and the use of topical steroids [9, 13]. Pharmaceutical therapy does not prevent the incidence of the chronic GVHD [14]. On the other hand, there is no certain treatment for GVHD, and the efficacy of the treatments seems to be limited [2]. Since 1980, corticosteroids have been used as the first line of treatment for oral GVHD. Moreover, immunosuppressants such as *cyclosporine* are used for the treatment of the chronic oral GVHD [15, 16]. The treatment of GVHD may cause complications such as secondary infection, hyperglycemia, hypertension, osteoporosis, renal insufficiency, and hyperlipidemia [17].

Treatment with curcumin has been successful for conditions such as rheumatoid arthritis, postsurgical inflammation, idiopathic orbital inflammation, Alzheimer’s disease, multiple myeloma, pancreatic cancer, colon cancer, lichen planus, and mucositis [16–23]. Several studies have demonstrated the anti-inflammatory effects of curcumin [24, 25]. It also inhibits apoptosis [26] and has protective effects against organ dysfunction, tissue damage, oxidative stress, and inflammation in response to ischemia. Moreover, it has antioxidant properties, it prevents lipid peroxidation and protects cellular macromolecules such as the DNA. Furthermore, it plays a role in the free oxygen radical scavenging activity [26–29].

To the best of our knowledge, no previous study has assessed the therapeutic efficacy of curcumin in the patients presenting with oral GVHD. Since curcumin has extensive therapeutic, anti-inflammatory, antimicrobial, and antioxidative effects and is nontoxic in its topical form, and by considering the side effects of the medications currently used for the treatment of GVHD [30, 31], we aimed to compare the efficacy of topical treatment with curcumin and triamcinolone in the patients affected by oral GVHD.

## MATERIALS AND METHODS

This study has been approved by the Ethics Committee of Tehran University of Medical Sciences and is registered at www.irct.ir (IRCT 201508173144N5). All the patients signed informed consent forms prior to participation in the study. Twenty-six patients presenting with oral GVHD and referring to Shariati Hospital of Tehran University of Medical Sciences were evaluated.

The inclusion criteria consisted of affliction with oral GVHD for a minimum of three months and no use of systemic medications for the treatment of oral lesions in the past three months. The diagnosis of oral GVHD was made and confirmed by an oral medicine specialist and an oncologist.

For the preparation of curcumin in Orabase, 500g of fresh curcumin powder was mixed with 2500ml of 75% ethanol, and the mixture was stored at room temperature overnight. The extract was collected, filtered, and distilled by vacuum distillation to obtain a resin mixture, which was refrigerated. Next, 100g of sodium carboxymethyl cellulose was filtered and added to 900ml of boiling water and was mixed by a magnetic mixer to obtain a stable gel. Afterwards, 10g of the extract was transferred to a 1000-ml beaker glass; 990g of the gel was also added, and the mixture was vigorously stirred for four hours and was transferred to test tubes.

Using block randomization, the patients were divided into two groups of 13. The control group received triamcinolone in Orabase, while the case group used curcumin in Orabase. The clinician was blinded to the group allocation of the patients. The medications were delivered in similar packages and had the same color and form. They were coded using the Microsoft Excel software program. The duration of the study was 28 days [6]. A visual analog scale (VAS) was used to assess the level of the pain severity. The GVHD scoring provided by the NIH was used to assess the severity of the lesions [12]. During the 28-day period, the patients were visited weekly to assess the treatment outcomes. In each session, the changes were determined based on the above-mentioned criteria.

### Statistical analysis:

Data were analyzed using SPSS version 22 software program (IBM SPSS Inc., Chicago, IL, USA). P<0.05 was considered statistically significant. The pain scores (according to the VAS) were compared using Mann-U-Whitney test, while the severity of the lesions was analyzed using the covariance test.

## RESULTS

**Fig. 1:**
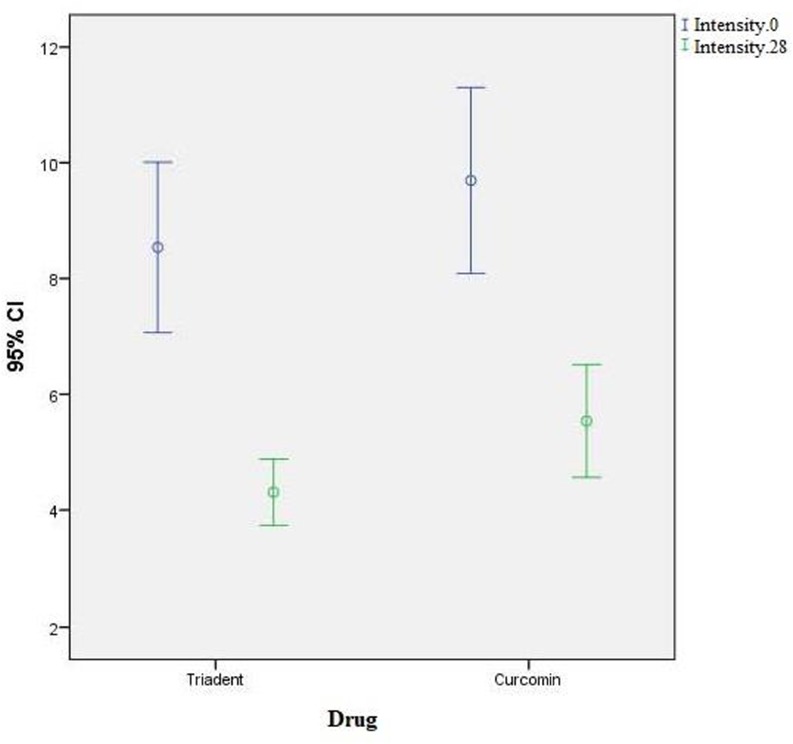
The error bar of the mean and 95% confidence interval (CI) of the severity of the oral mucosal involvement (mm^2^) at the baseline and after the treatment in the two groups

Thirteen cases and 13 controls were selected from among the patients presenting to the Hematology and Bone Marrow Transplantation Department of Shariati Hospital during 2015–2016. Table 1 shows the demographic characteristics of the patients. The case and control groups were not significantly different in terms of the malignancy type. All the patients had systemic GVHD and were under treatment with *prednisolone* and *cyclosporine*. The severity of the oral mucosal involvement (mm^2^) was compared between the two groups at the baseline and after the treatment. The statistical analysis showed that the mean severity in the case group (curcumin in Orabase) was 9.69±2.65mm^2^ at the baseline and 5.54±1.61mm^2^ after the intervention (Fig. 1).

**Table 1.** Demographic characteristics of the patients

	**Case**				**Control**			

**Sex**	**Male**		**Female**		**Male**		**Female**	

	8		5		7		6	
**Type of malignancy**	**AML**	**ALL**	**MM**	**Hodgkin’s lymphoma**	**AML**	**ALL**	**MM**	**Hodgkin’s lymphoma**
	9	2	2	0	7	2	3	1
**Age (years)**	**Minimum**	**Maximum**		**Mean±SD**	**Minimum**	**Maximum**		**Mean±SD**
	27	49		35.23±7.67	20	58		39.15±12.13

AML=Acute Myeloid Leukemia, ALL=Acute Lymphoblastic Leukemia, MM=Multiple Myeloma, SD=Standard Deviation

The mean severity in the control group (triamcinolone in Orabase) was 8.54±2.43mm^2^ at the baseline and 4.31±0.94mm^2^ after the intervention (Fig. 1).

The comparison of the alleviated severity between the two groups at the end of the treatment showed that the two groups were not significantly different (P=0.052).

**Fig. 2:**
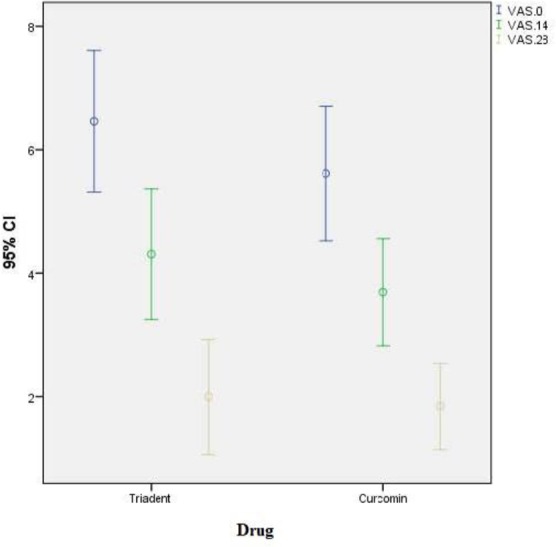
The error bar of the mean and 95% confidence interval (CI) of the severity of the pain (VAS) in the two groups at different time points

The comparison of the pain severity by the VAS at the baseline and at days 14 and 28 (completion of the treatment) between the two groups revealed that the mean pain score in the case group was 5.62±1.80 at the baseline, 3.69±1.43 at day 14, and 1.85±1.14 at day 28. The mean pain score in the control group was 6.46±1.89 at the baseline, 4.31±1.75 at day 14, and 2±1.52 at day 28 (Fig. 2).

The severity of the pain at the baseline (P=0.287), day 14 (P=0.362), and day 28 (P=0.687) was not significantly different between the two groups.

## DISCUSSION

Allogeneic hematopoietic stem cell transplantation (HSCT) is extensively used for the treatment of hematologic diseases. GVHD is among the most important and most serious complications of HSCT, which is associated with a high morbidity and mortality rate [2]. GVHD involves several organs and compromises the success of HSCT. It also decreases the life expectancy of the patients [14]. In the past couple of years, the prevalence of acute GVHD has remained constant, affecting 50 to 70% of the patients receiving allogeneic grafts. However, the prevalence of chronic GVHD has increased, as 30 to 50% of the patients who have received allogeneic grafts develop GVHD [8, 32]. There is no specific treatment for GVHD, and the efficacy of different treatment modalities is limited. Renal insufficiency, hypertension, hyperglycemia, secondary infection, osteoporosis, and hyperlipidemia are among the complications of GVHD [17]. Half of the patients with chronic GVHD, who have responded well to therapy, show reactivation of GVHD. This reactivation is correlated with the severity of the disease [33].

Systemic treatment is used for severe GVHD, as it involves several organs. The systemic treatment accompanied by local and topical treatments can be used for the patients with oral cavity or skin involvements. Corticosteroids are the medication of choice for the treatment of GVHD and can be used with or without the calcineurin inhibitors [2, 4, 11, 15]. In case of no response to the treatment with corticosteroids and in resistant cases, the second best choice would be extracorporeal *photopheresis* and also *sirolimus*, *everolimus*, *pentostatin*, and *rituximab* [4]. The drugs used for the treatment of GVHD, such as corticosteroids, have several side effects [17]. Some organic compounds including various spices and extracts are used in traditional medicine for wound healing and pain relief. They are rich sources of complex compounds with optimal therapeutic properties and can be effectively used for the treatment of oral GVHD [34, 35].

Curcumin is the main active constituent of *turmeric*, derived from the rhizomes (underground stems) of *Curcuma longa* from the family of *Zingiberaceae* [36]. In addition to curcumin (77%), other curcumin-like compounds known as curcuminoids (3%) are also available, including demethoxycurcumin and bisdemethoxycurcumin [37].

However, the pharmaceutical efficacy of *turmeric* powder is due to the presence of 77% curcumin. Curcumin has long been used in Southeast Asia as a spice and also for wound healing and pain and inflammation relief. The efficacy of this compound for the treatment of allergy, dermal wounds, stomachache, jaundice, bloody diarrhea, and chronic diseases such as cancers, neural conditions, cardiovascular conditions, and psychological problems has long been emphasized in traditional medicine. It does not have any significant side effects even at high doses. Such beneficial effects for a material in vitro and in vivo indicate that it can affect several cellular biochemical pathways [37, 38]. Due to these advantages, many studies have focused on curcumin and its pharmaceutical potential. Recent studies have shown that curcumin has several biological properties. It inhibits 5-lipooxygenase (5-LO) and decreases the level of tumor necrosis factor-alpha (TNF-α), interleukin-1beta (IL-1β), and interferon-gamma (IFN-γ) cytokines and exerts anti-inflammatory [24, 25] and antioxidative [39, 40] effects. Oxidative stress and TNF-α play a role in the emergence and aggravation of GVHD [41]; this explains the improvement noticed in the curcumin group in our study. Curcumin also has antibacterial, antifungal, antiviral, and disinfecting properties. Evidence shows that secondary infection with Candida albicans or alterations in the oral microbial flora can aggravate the oral lesions in GVHD [41]. Thus, due to its antifungal and antibacterial properties, curcumin may be preferred to the typical steroid therapy for the management of GVHD. Also, curcumin inhibits the transforming growth factor-beta (TGF-β) and Caspase-3 and prevents apoptosis [26]. Several studies have shown that curcumin has protective effects against tissue damage, oxidative stress, and inflammation in response to ischemia [26, 29].

Cancer is among the long-term complications of oral GVHD [42]. Carcinogenesis is a multistep process, in which different biochemical pathways and the function of many cellular proteins such as transcription factors, cytokines, enzymes, cell-proliferation regulating genes, and apoptosis genes are impaired. Curcumin, with its anticancer properties, affects many pathways and the related proteins [21, 29]. In addition, the anticancer property adds to the superiority of curcumin treatment compared to conventional treatments.

Curcumin regulates the pro-angiogenic growth factors such as basic fibroblast growth factor (bFGF) and vascular endothelial growth factor (VEGF), transcription enzymes, angiopoietin-1 and -2, cyclooxygenase-2 (Cox-2), and matrix metallopeptidase-9 (MMP-9) [43–45]. It also has inhibitory effects on bFGF (an angiogenic stimulator). In addition, it down-regulates the expression of the MMP-9 and enzymes required for angiogenesis [44]. In conclusion, curcumin has extensive anti-inflammatory, antimicrobial, and antioxidative properties, is nontoxic when applied topically, and has optimal efficacy for the treatment of rheumatoid arthritis [15], post-surgical inflammation [16], idiopathic orbital inflammation [18], Alzheimer’s disease, multiple myeloma [19], pancreatic and colon cancers [21, 22], and oral mucositis, as shown by Mansourian et al [20].

Thus, we compared the efficacy of curcumin and triamcinolone in the patients presenting with oral GVHD [19, 20] and showed that the two groups of patients subjected to curcumin in Orabase and triamcinolone in Orabase were not significantly different in terms of the pain (VAS) and severity of the lesions. The anti-inflammatory properties of curcumin, including the inhibitory effect on 5-LO and reducing the level of TNF-α, IL-1β, and INF-γ [24, 25] are probably the reason for the equal efficacy of triamcinolone (which also has anti-flammatory effects) and curcumin. Also, by considering the antioxidative properties [39, 40] and protective effects against tissue damage and oxidative stress in the treatment of GVHD, curcumin can be used for the patients affected by oral GVHD since it poses fewer complications compared to the currently used drugs.

## CONCLUSION

Curcumin in Orabase has comparable efficacy to that of triamcinolone in Orabase and may be beneficial for the treatment of the patients presenting with oral GVHD.
